# Robotic Stereotactic Body Radiation Therapy for High-Risk Prostate Cancer: The Georgetown University Experience

**DOI:** 10.7759/cureus.85798

**Published:** 2025-06-11

**Authors:** Vaibhav Sharma, Tim Kearney, Zach Lee, Padraig Brennan Pilkington, Marielle Fis Loperena, Malika Danner, Alan L Zwart, Deepak Kumar, Brian Collins, Michael Carrasquilla, Suy Simeng, Sean Collins

**Affiliations:** 1 Department of Radiation Medicine, MedStar Georgetown University Hospital, Washington, D.C., USA; 2 Department of Radiation Medicine, Georgetown University School of Medicine, Washington, D.C., USA; 3 Department of Radiation Medicine, George Washington University School of Medicine and Health Sciences, Washington, D.C., USA; 4 Department of Radiation Medicine, University of South Florida (USF) Health Morsani College of Medicine, Tampa, USA; 5 Department of Oncology, Lombardi Comprehensive Cancer Center, Washington, D.C., USA; 6 Department of Radiation Oncology, Julius L. Chambers Biomedical/Biotechnology Institute, North Carolina Central University, Durham, USA

**Keywords:** biochemical recurrence, bowel function, high-risk prostate cancer, hypofractionation, long-term effects, quality of life, stereotactic body radiotherapy, urinary function

## Abstract

Introduction

Stereotactic body radiation therapy (SBRT) has emerged as a highly conformal and hypofractionated treatment modality, demonstrating safety and efficacy in low- and intermediate-risk prostate cancer (PCa). Traditionally, high-risk (HR) PCa has been managed with conventional fractionation external beam radiotherapy. Such extended treatment may be burdensome to elderly PCa patients. There is a dearth of long-term patient-reported outcome data for HR PCa patients treated with SBRT. This retrospective study examines cancer control and health-related quality of life (HRQOL) outcomes in HR PCa patients receiving robotic SBRT.

Materials and methods

HR PCa patients who underwent robotic SBRT treatment (7-7.25 Gy in five fractions over one to two weeks) from December 2008 to July 2023 were included in this retrospective analysis. Biochemical failure was defined according to the Phoenix criteria as a rise in PSA of ≥2 ng/mL above the nadir. Patterns of failure were classified as PSA only, local, pelvic node, abdominal node, or bone. Patients completed the 26-item expanded PCa index composite (EPIC)-26 questionnaire at baseline, three, six, 12, 18, 24, and 36 months post radiotherapy. HRQOL domain scores for urinary incontinence, urinary irritative/obstructive, and bowel function were calculated following EPIC-26 scoring guidelines, with higher scores indicating improved quality of life (QOL). Kruskal-Wallis tests and Post-Hoc Dunn Multiple Comparison Tests were employed to examine significant changes within HRQOL domains. Minimally important differences were calculated using 0.5 of a standard deviation at baseline.

Results

A total of 216 patients, with a median age of 75 years, completed the treatment and had a median follow-up of 40 months. Seventy-five percent of patients received androgen deprivation therapy prior to radiotherapy initiation. The three-year biochemical disease-free rate was 89%. Among all recurrences, bone metastases were the most common (34.15%), followed by PSA-only recurrences (24.39%), local recurrences (17.08%), and abdominal and pelvic lymph node involvement (12.2% each). At the initiation of RT, patients exhibited a urinary incontinence domain score of (mean ± SD) 86.04 ± 1.27, a urinary irritative/obstructive domain score of 83.4 ± 1.06, and a bowel domain score of 92.7 ± 0.85. Three years post-treatment, the urinary incontinence domain score decreased to 84.4 ± 1.9, the urinary irritative/obstructive domain score increased to 86.3 ± 1.34, and the bowel domain score decreased to 90.63 ± 1.37. These changes did not reach statistical and/or clinical significance.

Conclusions

At the three-year follow-up mark, favorable cancer control was achieved, and patients had recovered mainly to near baseline urinary and bowel function. SBRT demonstrated excellent tolerability with minimal impact on PCa-specific HRQOL in HR PCa patients. These findings underscore the potential of SBRT as a convenient treatment option for HR PCa, offering promising outcomes and preserving patient QOL.

## Introduction

Stereotactic body radiation therapy (SBRT) in low-risk (LR) and intermediate-risk (IR) prostate cancer (PCa) has demonstrated excellent tumor control with acceptable toxicity [1-3]. Two randomized trials comparing conventionally fractionated versus SBRT revealed comparable rates of physician-reported toxicities and patient-reported outcomes, emphasizing the consistent safety profile of SBRT [4,5]. Five-year biochemical failure-free rates were high in participants who received SBRT and were non-inferior to those of conventional radiotherapy [4,5]. These excellent results have established SBRT as the standard of care for LR and IR PCa patients [6].

The role of SBRT in high-risk (HR) PCa patients is an area of active clinical investigation. Conventionally fractionated radiation therapy (RT) for HR PCa has historically yielded poor cancer control [7]. Dose escalation via brachytherapy or SBRT boost has been utilized in the HR population, resulting in improved biochemical disease-free survival (BDFS), but with increased high-grade toxicity, inconvenience, and cost [8-11]. Androgen deprivation therapy (ADT) in combination with RT is recommended in HR patients to improve cancer control [6]. Results from the SHARP consortium showed improved BDFS in HR patients treated with SBRT receiving ADT with estimated four-year biochemical recurrence-free survival and distant metastasis-free survival rates of 82% and 89%, respectively [4]. The incidence of late grade 3 or higher genitourinary and gastrointestinal complications was low at 2.3% and 0.9%, respectively [4].

Robotic SBRT delivers hundreds of individualized non-isocentric beams with a targeting error of less than 1 mm, allowing the safe delivery of highly conformal treatment plans with steep dose gradients [12,13]. Unlike standard image-guided RT, robotic SBRT incorporates a real-time tracking system that provides updated prostate position information to the robot, allowing it to correct the targeting of the therapeutic beam during treatment [14]. This feature enables a reduction in the planning target volume (PTV), thereby better limiting the dose to surrounding critical organs. Recent analysis suggests that this approach may allow for intra-prostatic dose escalation with reduced urinary toxicity [15]. Here, we present our institutional experience with robotic SBRT for HR PCa.

## Materials and methods

Patient selection

Patients eligible for inclusion in this retrospective study were those with HR PCa, as classified by D’Amico, who underwent robotic SBRT (CyberKnife, Accuray, Madison, WI) at Medstar Georgetown University Hospital from December 2008 to July 2023 [16]. Exclusion criteria included distant metastasis at baseline, prior pelvic radiotherapy, and/or prior radical prostatectomy. The Georgetown University Institutional Review Board approved this single institutional retrospective review (approval number: 2009-510).

Stereotactic body radiation therapy

Robotic SBRT was delivered as previously described [13,17]. Three to six gold fiducial markers were placed in the prostate. Seven days later, a treatment planning MRI was obtained, followed by a non-contrast simulation CT scan with 1.25 mm slice thickness. Both scans were done with an empty bladder, and patients were advised to have a low-gas, low-motility diet at least five days prior to imaging and treatment delivery. Patients did not take anything orally the night before the simulation, and an enema was administered one to two hours before imaging and treatment. Fused MR and CT scans were then used for treatment planning. The clinical target volume included the prostate, areas of radiographic extracapsular extension, and proximal seminal vesicles to the point where the left and right seminal vesicles separate. The rectum, bladder, and membranous urethra were contoured and evaluated. The SBRT-PTV equaled the clinical target volume, spaced 5 mm to the right and left and 3 mm elsewhere. Patients were treated with an SBRT prescription dose of 35-36.25 Gy to the PTV, which was delivered in five fractions of 7-7.25 Gy. Target position was verified multiple times during each treatment using paired, orthogonal X-ray images with a minimum of three properly placed fiducials. Shared decision-making was utilized to determine the use and duration of ADT [18].

Pretreatment assessment, follow-up, and statistical analysis

Prostate-specific antigen (PSA) levels were obtained, and PCa-specific QOL questionnaires were administered before the first SBRT treatment (baseline) and at three months, six months, 12 months, 18 months, 24 months, and then yearly after completion of RT. PSA nadirs were defined as the lowest PSA prior to failure. If a PSA rose, a digital rectal exam (DRE) was performed, and the best available imaging at the time was obtained as previously described [19]. Imaging studies, such as bone scans, abdominal and pelvic CT scans, and, more recently, PET imaging, were used to identify distant failures. Patterns of failure were classified as PSA only, local, pelvic node, abdominal node, or bone. Biochemical failure was classified as PSA-only if no malignancy was seen on the scan. Local failure was classified as occurring only in the prostate. Lymph node failure was classified as pelvic or abdominal. If bone metastases were identified, the failure was classified as bone independent of nodal status.

The health-related quality of life (HRQOL) domain scores for urinary incontinence, urinary irritative/obstructive, and bowel function were determined by the expanded PCa index composite (EPIC)-26 quality of life (QOL) questionnaire as previously described [20]. Briefly, EPIC scores ranged from 0 to 100, and higher scores indicated improved QOL. Differences in ongoing QOL scores were assessed and compared to the baseline and each follow-up using the Kruskal-Wallis test. This test was used to determine the significance of the difference between nonparametric, ordinal data. The Post-Hoc Dunn Multiple Comparison Test was employed to examine significant changes within HRQOL domains. Minimally important differences were calculated using 0.5 of a standard deviation at baseline.

## Results

Patients

Between December 2008 and July 2023, 216 HR PCa patients were treated with robotic SBRT. The median follow-up was 40 months. Table 1 provides a summary of patient characteristics. The median age was 75 years (range: 54-94). Caucasian patients comprised 50% of the subjects, while Black patients accounted for 39% of the subjects. The median pretreatment PSA was 14.2 ng/ml (range: 1.3-148 ng/ml). The median prostate volume was 39 cc (range: 15-186 cc). The median baseline Charleston Comorbidity Index (CCI) score was 1, with 9.7% of patients having a CCI score above 3. ADT was administered to 75% of patients for a median duration of 10 months (range: 3-48 months).

**Table 1:** Patient characteristics PSA: prostate-specific antigen

Domain	Patients (N=216)		Percent patient (%)
Age			
<60	7		3
60-69	53		25
70-79	84		39
>80	71		33
Race			
White	107		50
Black	84		39
Hispanic	4		2
Other	21		10
Initial PSA			
<10	82		38
10-20	50		23
>20	84		39
Gleason score			
3 + 3 = 6	19		9
3 + 4 = 7	26		12
4 + 3 = 7	23		11
4 + 4 = 8	101		47
3 + 5 = 8	8		4
4 + 5 = 9	37		17
5 + 5 = 10	2		1
Dose			
35 Gy/5 fx	38		18
35.5 Gy/5 fx	2		1
36.25 Gy/5 fx	176	81	
Hormone			
Yes	156		75
No	51		25

Cancer control

**Figure 1:**
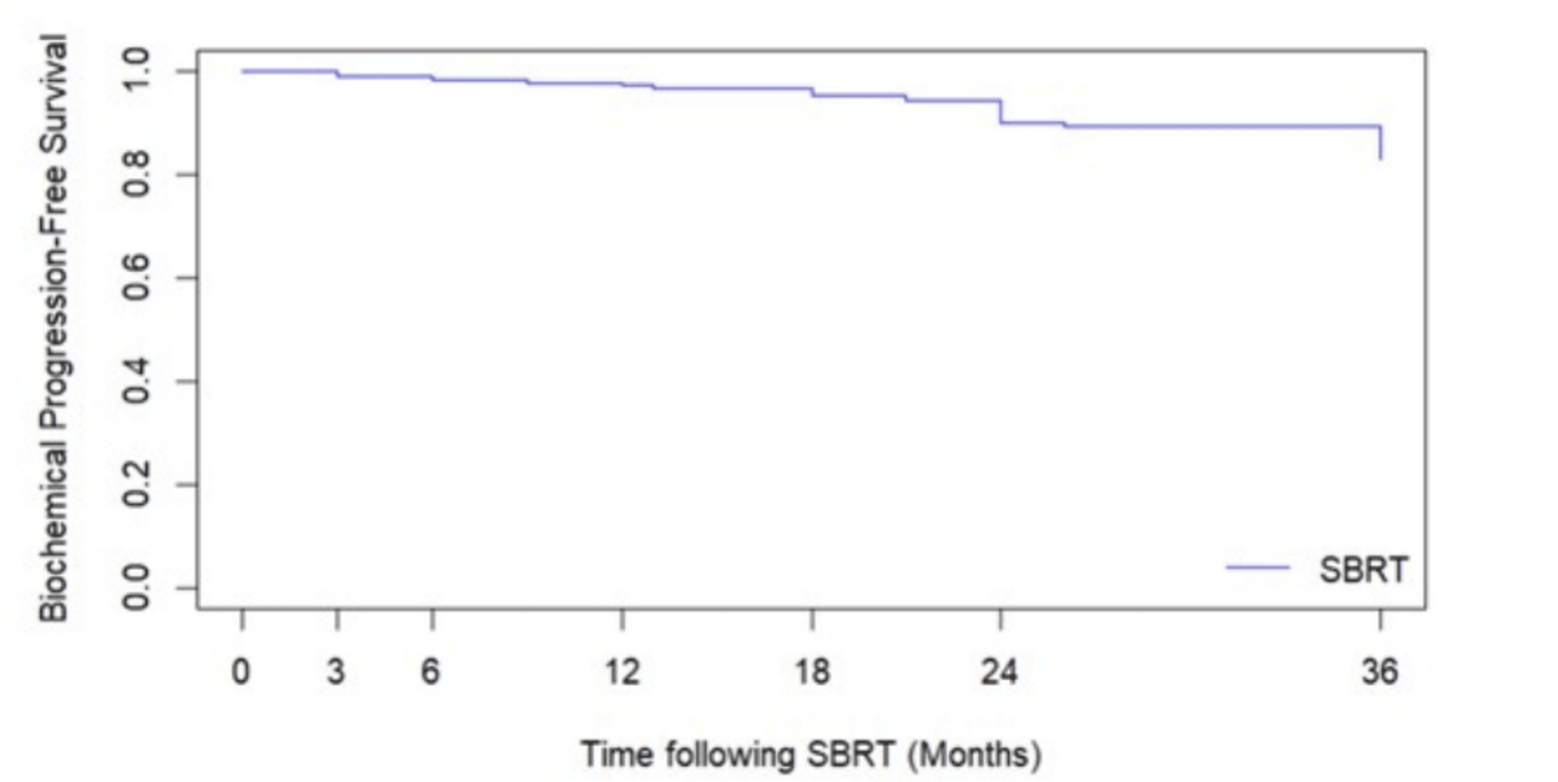
Biochemical control for HR PCa receiving SBRT at three-year follow-up SBRT: stereotactic body radiation therapy, HR PCa: high-risk prostate cancer

In our analysis, a total of 33 recurrences (15% of total subjects) were observed, with 23 (11% of total subjects) occurring within 36 months, indicating a BDFS rate of 89% at three years. Figure 1 shows the BDFS curve, and Table 2 provides the patient characteristics of those who failed treatment. Notably, four recurrences (12% of all recurrences, 2% of total subjects) occurred within the first 12 months (Table 3). The median time to failure was 36 months, with a range of 3 to 121 months. The average initial PSA was 22.9 ng/ml, with an average PSA nadir of 1.82 ng/ml post-treatment and an average PSA at recurrence of 22.03 ng/ml.

**Table 2:** Characteristics of failure PSA: prostate-specific antigen, DRE: digital rectal exam

	Average (range)
PSA ng/ml	
Initial	22.9 (1.3-6)
Nadir	1.82 (0.1-34)
Recurrence	22.03 (0.39-429.8)
Time to failure (months)	40 (3-131)
	Percent patients N (%)
Gleason score	
G6	1 (3%)
G7	8 (24%)
G8	12 (36%)
G9	12 (36%)
Dose (Gy)	
35	6 (18%)
36.25	27 (82%)
DRE	
Abnormal	18 (55%)
Normal	15 (45%)
Pattern of failure	
Bone	11 (34.15%)
Local	6 (17.08%)
PSA only	8 (24.39%)
Abdomen	4 (12.2%)
Pelvis	4 (12.2%)

**Table 3:** Clinical characteristics of patients who experienced PSA recurrence after being treated with SBRT PSA: prostate-specific antigen, SBRT: stereotactic body radiation therapy, ADT: androgen deprivation therapy, DRE: digital rectal exam, BS: bone scan, MRI: magnetic resonance imaging, CT: computed tomography, PET: positron emission tomography, PSMA: prostate-specific membrane antigen

ID	iPSA	Stage	Gleason	Staging imaging	Dose	ADT	PSA nadir	Time to failure	PSA recurrence	DRE	Recurrence imaging	Pattern of failure	Radiotherapy
1	50	T3a	G7	BS, MRI	36.25	Y	0.1	24	5.6	Abnormal	BS, CT	Abd	N
2	20	T2c	G9	MRI	35	N	1.7	36	3.8	-	-	PSA only	N
3	31.8	T2a	G6	BS, CT, MRI	36.25	Y	0.6	24	3.9	Normal	BS	Bone	Curative
4	32.5	T1c	G7	MRI	36.25	N	0.5	96	3.2	Normal	Choline, C-11, PET, axumin	Abd	N
5	38	T2b	G9	BS, MRI	36.25	Y	0.35	24	3.94	Normal	-	PSA only	N
6	15.5	T2b	G9	BS, CT, MRI	36.25	Y	0.1	48	3.0	Abnormal	Axumin, MRI, PSMA	Bone, local	N
7	20.4	T1c	G7	MRI	36.25	N	6.8	9	11	Normal	BS, CT	Bone	Curative
8	47	T2	G9	BS, CT	36.25	Y	<0.1	36	5.1	Abnormal	PSMA	Local	N
9	6.8	T2c	G8	-	35	Y	0.1	24	4	Abnormal	-	PSA only	N
10	8.4	T2a	G8	-	36.25	Y	0.5	21	4.8	Normal	BS	Bone	Curative
11	54	T2b	G9	MRI	36.25	N	0.9	13	2.8	Abnormal	BS, CT	Bone, pelvis	N
12	12.6	T1c	G8	BS, CT, MRI	36.25	Y	0.1	24	2.3	Abnormal	BS, CT	PSA only	N
13	1.3	T2a	G8	MRI	36.25	N	1.23	48	2.6	Abnormal	Axumin	Abd, bone, pelvis	Curative
14	7.9	T2b	G8	-	36.25	Y	0.6	18	3.1	Abnormal	Biopsy, BS, MRI	Bone, pelvis	Palliative
15	25.4	T2a	G7	CT	36.25	Y	2.2	36	24	Normal	BS, MRI	Bone, local	Curative
16	10.3	T2b	G9	BS	35	Y	0.1	36	2.2	Abnormal	-	PSA only	N
17	40	T2c	G8	BS	36.25	Y	<0.1	18	3.9	Abnormal	BS	PSA only	N
18	17	T2b	G8	-	35	N	0.9	42	2.9	Normal	BS	Bone	N
19	8.3	T2c	G7	MRI	36.25	N	0.22	131	4.3	Abnormal	Axumin	PSA only	N
20	60	T2b	G7	MRI	36.25	Y	3.6	36	55.7	Normal	MRI	Local	N
21	31.6	T1c	G7	BS, MRI	36.25	Y	0.1	90	429.8	Normal	BS, CT	PSA only	N
22	46.1	T2b	G8	BS, CT, MRI	35	Y	34	3	61.9	Abnormal	BS	Bone	Palliative
23	5.5	T1c	G9	MRI	36.25	Y	0.1	50	5.27	Normal	MRI, CT, BS	Bone, local	N
24	8.5	T2c	G9	BS	36.25	Y	1.6	3	6.2	Abnormal	BS, CT	Bone	N
25	4.3	T1c	G8	CT, MRI	36.25	Y	<0.1	36	36.6	Normal	BS, PSMA	Abd, pelvis	N
26	4.7	T2a	G9	BS, CT	36.25	Y	<0.1	104	0.39	Normal	PSMA	Abd	Curative
27	37.5	T2a	G8	BS, MRI	36.25	N	0.8	80	3.1	Normal	MRI, PSMA	Local	N
28	17.6	T1c	G9	MRI	36.25	N	1	64	3.2	Normal	MRI	Local	N
29	20.3	T2c	G9	-	35	Y	0.8	24	8.8	Abnormal	BS	Bone	No
30	4.8	T1b	G9	BS, MRI	36.25	Y	0.4	26	2.9	-	PSMA	Pelvis	Curative
31	22.4	T2c	G8	BS, CT	36.25	Y	0.1	6	6.3	Abnormal	BS	Bone	N
32	38	T2b	G7	BS	36.25	Y	0.2	36	5.1	-	-	PSA only	N
33	7.9	T1c	G8	BS	36.25	Y	<0.1	56	5.2	Normal	MRI	PSA only	N

Recurrence characteristics

**Figure 2:**
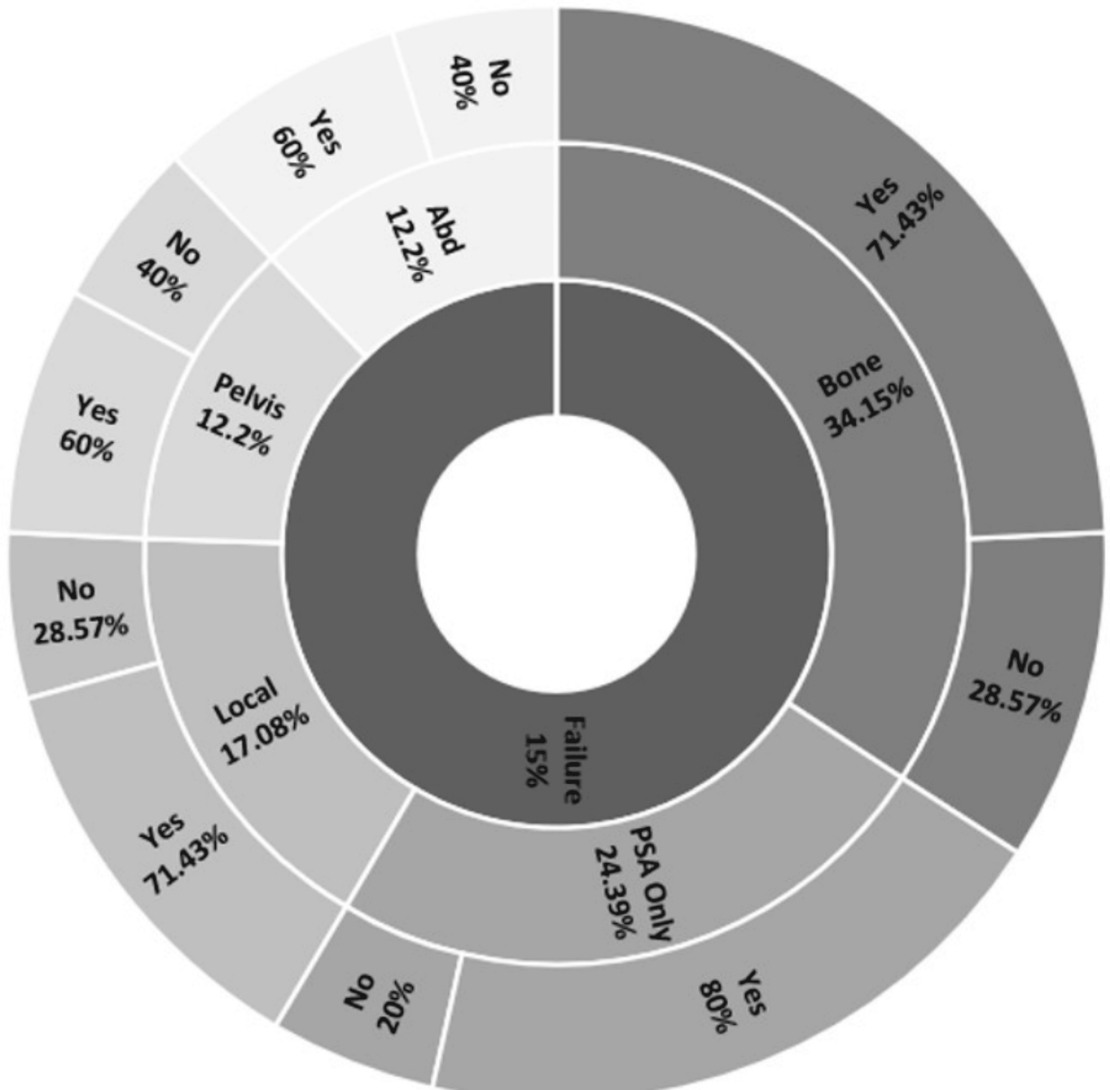
Failure patterns after SBRT in HR PCa Types of failure in HR PCa after five-fraction SBRT, alongside ADT use (yes/no) for each failure type SBRT: stereotactic body radiation therapy, HR PCa: high-risk prostate cancer, ADT: androgen deprivation therapy, PSA: prostate-specific antigen

Among all recurrences, bone metastases were the most common (34.15%), followed by PSA-only recurrences (24.39%), local recurrences (17.08%), and abdominal and pelvic lymph node involvement (12.2% each) (Table 2, Figure 2). There were no isolated seminal vesicle recurrences. High-grade disease was common, with Gleason scores of 8 and 9 accounting for 36% of recurrences each, Gleason 7 in 24%, and Gleason 6 in only 3%. ADT was used in 70% of recurrent cases, while 30% did not receive ADT. On clinical examination during recurrence follow-up, 47% had normal DRE findings and 53% had abnormal DREs.

Quality of life

**Figure 3:**
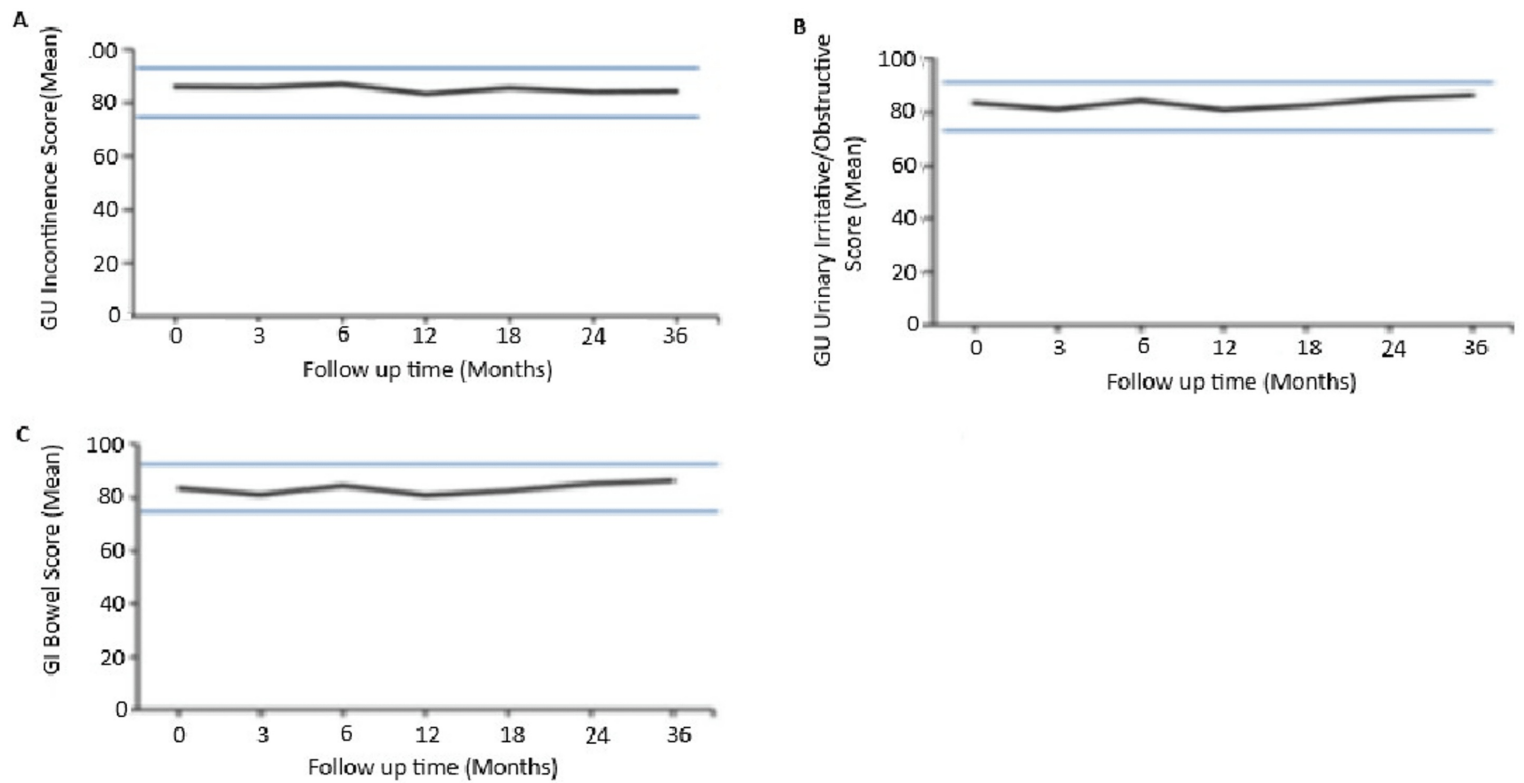
Average EPIC domain scores at baseline and follow up for HR PCa patients after receiving SBRT (A) EPIC GU incontinence domain. (B) EPIC GU irritative/obstructive domain. (C) EPIC bowel domain. The upper and lower thresholds represent clinically significant changes in score ( ½ SD). EPIC scores range from 0 to 100, with higher values indicating a more favorable QOL. EPIC: expanded prostate cancer index composite, SBRT: stereotactic body radiation therapy, GU: genitourinary, QOL: quality of life, SD: standard deviation, HR PCa: high-risk prostate cancer

Figure 3 shows QOL following robotic SBRT. The study found that the mean urinary incontinence function score decreased slightly from 86.04 at baseline to 84.4 at the 36-month follow-up (p>0.05). Weak stream (14%) and frequency (24%) were the most common symptoms in the urinary irritative/obstructive domain. Similarly, the mean bowel function score showed a minor reduction from 92.7 at baseline to 90.6 at 36 months, without statistical significance (p>0.05). Bowel symptoms were less frequent than urinary symptoms, with urgency ranging from 5% to 7% over the study period, peaking at 7% at six months. The percentage of patients reporting bowel frequency increased from 3% at baseline to a peak of 8% at 18 months.

## Discussion

This study aimed to assess failure patterns and QOL metrics in a cohort of HRPC patients who received robotic SBRT without elective pelvic irradiation. Our results add to the growing body of evidence that definitive prostate SBRT for localized HRPC may have similar outcomes for patients while sparing them elective pelvic radiation dose. Our study saw favorable local disease control, with a three-year BDFS of 89%. These numbers are consistent with those of the SHARP consortium, which assessed a group of HRPC patients who also underwent definitive prostate SBRT [21]. We found that local failures constituted 21% of the total three-year recurrences, suggesting that BDFS could be higher with higher prescription doses. A growing body of evidence indicates that dose escalation to the DIL, or the prostate as a whole, significantly improves local control, and our findings support these data [22-24]. Staging PSMA-PET scans, with their emerging prominence in diagnosing and localizing HR primary PCa and recurrent disease, could help us safely escalate doses for our patients.

Pelvic lymph node recurrence constituted only 15% of our failures. This suggests elective nodal irradiation (ENI) would not have improved the clinical outcomes of most of our cohort by the three-year follow-up. Indeed, the literature regarding elective pelvic ENI remains controversial, showing mixed benefits [25]. Additionally, those who undergo whole-pelvis radiotherapy (WPRT) usually experience much higher rates of gastrointestinal toxicity in the long term. The POP-RT trial shows improved short-term BDFS with WPRT; however, most of these patients underwent pre-treatment PSMA imaging to rule out metastases or locoregional spread [26]. Our patients did not undergo PSMA imaging during initial staging, so it is difficult to apply the POP-RT results to our cohort. It is possible that had our patients undergone PSMA imaging prior to treatment, pelvic nodal metastases could have been identified, in which case these would not be considered failures from the omission of pelvic ENI. Furthermore, abdominal lymph node recurrence constituted 12.2% of our total failures, a proportion that is favorably small. These failures would likely not have been impacted by WPRT.

Our cohort had a relatively large proportion of patients from lower socioeconomic backgrounds, who faced numerous social challenges. Many of our patients had difficulty with transportation and social support. A large proportion were frail. These patients likely would have had difficulty with a several-week regimen of daily RT. Our results can therefore be applied to these populations, demonstrating that five-fraction SBRT remains a highly favorable option, even for HR PCa. Recent data suggest that ultrahypofractionated elective pelvic nodal irradiation (25 Gy in five fractions) is safe and effective at preventing pelvic nodal recurrences [27]. The inability to treat the prostate and pelvic nodes simultaneously is a known limitation of robotic SBRT [28].

Despite highlighting the favorable outcomes associated with hypofractionated SBRT in HR PCa patients, we acknowledge the limitations inherent in our retrospective study design and reliance on patient-reported outcomes. Notably, the absence of stratification and propensity-matched scoring warrants further exploration in future research to validate these findings. There was variability in initial imaging during the staging and workup of each patient, affecting cohort uniformity.

## Conclusions

Definitive prostate SBRT for localized HRPC has been shown to yield similar short-term oncologic outcomes to the current standard of care while still affording patients an excellent QOL. We see non-significant decreases in the gastrointestinal and genitourinary EPIC domain scores, suggesting that this treatment yields minimal long-term toxicity at three-year follow-up. We also note that only 12.2% of treatment failures occurred in the pelvis, prompting us to question the cost-benefit utility of electively irradiating the pelvis in the first place. Prostate SBRT spares this dose to the pelvis while still allowing the possibility for future salvage RT if locoregional failure occurs. While the idea of elective pelvic nodal irradiation remains controversial in the HR patient population, our data support local definitive treatment as a favorable option. This option should be especially considered for patients who are at a higher risk of missing treatments or who prefer a shorter course.
